# Intrinsic brain activity reorganization contributes to long-term compensation of higher-order hearing abilities in single-sided deafness

**DOI:** 10.3389/fnins.2022.935834

**Published:** 2022-08-25

**Authors:** Yufei Qiao, Min Zhu, Wen Sun, Yang Sun, Hua Guo, Yingying Shang

**Affiliations:** ^1^Department of Otorhinolaryngology, Peking Union Medical College Hospital, Beijing, China; ^2^School of Educational Science, Shenyang Normal University, Shengyang, China; ^3^Department of Biomedical Engineering, Center for Biomedical Imaging Research, School of Medicine, Tsinghua University, Beijing, China

**Keywords:** single-sided deafness, resting-state fMRI, intrinsic brain activity, speech recognition, sound localization, compensatory mechanism

## Abstract

Single-sided deafness (SSD) is an extreme case of partial hearing deprivation and results in a significant decline in higher-order hearing abilities, including sound localization and speech-in-noise recognition. Clinical studies have reported that patients with SSD recover from these higher-order hearing abilities to some extent over time. Neuroimaging studies have observed extensive brain functional plasticity in patients with SSD. However, studies investigating the role of plasticity in functional compensation, particularly those investigating the relationship between intrinsic brain activity alterations and higher-order hearing abilities, are still limited. In this study, we used resting-state functional MRI to investigate intrinsic brain activity, measured by the amplitude of low-frequency fluctuation (ALFF), in 19 patients with left SSD, 17 patients with right SSD, and 21 normal hearing controls (NHs). All patients with SSD had durations of deafness longer than 2 years. Decreased ALFF values in the bilateral precuneus (PCUN), lingual gyrus, and left middle frontal gyrus were observed in patients with SSD compared with the values of NHs. Longer durations of deafness were correlated with better hearing abilities, as well as higher ALFF values in the left inferior parietal lobule, the angular gyrus, the middle occipital gyrus, the bilateral PCUN, and the posterior cingulate gyrus. Moreover, we observed a generally consistent trend of correlation between ALFF values and higher-order hearing abilities in specific brain areas in patients with SSD. That is, better abilities were correlated with lower ALFF values in the frontal regions and higher ALFF values in the PCUN and surrounding parietal-occipital areas. Furthermore, mediation analysis revealed that the ALFF values in the PCUN were a significant mediator of the relationship between the duration of deafness and higher-order hearing abilities. Our study reveals significant plasticity of intrinsic brain activity in patients with SSD and suggests that reorganization of intrinsic brain activity may be one of the compensatory mechanisms that facilitate improvement in higher-order hearing abilities in these patients over time.

## Introduction

Single-sided deafness (SSD) is an extreme case of partial hearing deprivation and refers to severe to profound hearing loss in one ear and normal hearing in the other ear. Due to hearing deprivation in one ear, patients with SSD can only obtain monaural clues from the environment. This causes these patients to have sharply decreased higher-order hearing abilities, particularly sound localization and speech-in-noise (SIN) recognition (Agterberg et al., 2014; Asp et al., 2018; Liu et al., 2018; Adigun and Vangerwua, 2021). Studies have observed better hearing abilities in SSD patients with longer durations of deafness than in those with shorter durations of deafness, suggesting that functional compensation occurs over time (Peckham and Sheridan, 1976; Lieu et al., 2012; Liu et al., 2018). Since the peripheral auditory input in most patients with SSD could hardly be improved due to the irreversible property of sensorineural hearing loss and the lack of binaural clues remains unchanged, it could be conjectured that central plasticity promoting better usage of limited peripheral auditory input probably plays an important role in functional compensation.

To date, a growing number of neuroimaging studies have explored the central structural and functional plasticity that occurs due to SSD. Structural studies *via* magnetic resonance imaging (MRI) have observed extensive morphological alterations in gray and white matter, as well as structural connectivity involving auditory areas, other sensory areas, and higher-order cognitive-related brain areas (Lin et al., 2008; Wu et al., 2009; Rachakonda et al., 2014; Yang et al., 2014; Fan et al., 2015; Wang et al., 2016; Li et al., 2019). Regarding function, both functional MRI (fMRI) and event-related potential (ERP) studies using auditory stimuli have found that the auditory cortex shows a more symmetrical and synchronous response to monaural sound stimuli in patients with SSD than in individuals with normal hearing (NH) (Scheffler et al., 1998; Bilecen et al., 2000; Ponton et al., 2001; Khosla et al., 2003; Langers et al., 2005). Studies using visual or visual-audio tasks have revealed cross-modal plasticity in patients with SSD (Schmithorst et al., 2014; Qiao et al., 2019). Furthermore, functional alterations in brain regions related to higher-order cognitive function have been observed in auditory, visual, and visual-audio task studies (Schmithorst et al., 2014; Shang et al., 2018; Qiao et al., 2019). Compared with task-based studies examining task-related brain activity, an advantage of resting-state imaging approaches is that they allow the examination of intrinsic brain function in the absence of theory-driven tasks. Widespread resting-state functional connectivity alterations were observed in patients with SSD in brain regions and networks related not only to auditory processing but also to other sensory functions, such as vision, as well as higher-order cognitive control (Wang et al., 2014; Zhang et al., 2015, 2016, 2018a,b; Xu et al., 2016; Zhu et al., 2021). However, most of these studies did not investigate the relationship between central plasticity and higher-order hearing abilities in patients with SSD. Therefore, it is difficult to determine which of the above plasticity conditions contribute to auditory functional compensation in patients with SSD.

A previous fMRI study of children with unilateral sensorineural hearing loss performing SIN recognition tasks reported changes in activation in regions of the attention network, in addition to changes in secondary auditory processing areas and visual associated areas (Propst et al., 2010). However, this study did not investigate the correlation between brain activation and behavioral performance on the SIN recognition task. Therefore, this study does not provide reliable evidence that brain functional plasticity is compensatory for hearing abilities. Li's et al. diffusion tensor imaging (DTI) study revealed a strong correlation between SIN recognition ability and the strength of structural network connectivity, mainly in the frontoparietal regions, suggesting that the structural reorganization of cognitive-related networks may be one of the compensatory mechanisms (Li et al., 2019). However, to the best of our knowledge, no similar study has explored the relationship between functional reorganization and higher-order hearing abilities in SSD. Thus, the underlying mechanisms of compensation in SSD require further study.

The amplitude of low-frequency fluctuation (ALFF) of resting-state fMRI reflects the intensity of regional brain activity at baseline (Zang et al., 2007; Zou et al., 2008; Wang et al., 2011). ALFF has been widely used in studies of various neurological and sensory dysfunctional diseases, such as Alzheimer's disease (Wang et al., 2011; Mu et al., 2020; Yang et al., 2020), attention-deficit hyperactivity disorder (An et al., 2013; Jiang et al., 2020), high myopia, and monocular blindness (Huang et al., 2016; Fang et al., 2020). A previous study using ALFF investigated the alteration in intrinsic brain activity in patients with right-sided unilateral hearing loss and observed decreased ALFF values in the precuneus (PCUN), inferior parietal lobule (IPL), inferior frontal gyrus (IFG), and insula (INS) and increased ALFF values in the inferior temporal gyrus and middle temporal gyrus compared with the values of NHs (Yang et al., 2014). Furthermore, a positive correlation between disease duration and ALFF values was observed in certain brain regions, including the superior temporal gyrus, IFG, INS, and superior frontal gyrus (SFG) (Yang et al., 2014). These results suggest that ALFF is a promising biomarker of neurophysiological consequences that can indicate changes in regional signals of brain intrinsic activity. However, no study has used ALFF to explore the contribution of brain functional plasticity to the compensation of higher-order hearing abilities in patients with SSD.

The present study aimed to investigate the alteration in intrinsic brain activity in patients with long-term SSD and clarify the relationship among brain activity, duration of deafness, and higher-order hearing abilities. We used ALFF to investigate the alteration in intrinsic brain activity. We also evaluated the patients' higher-order hearing abilities, including sound localization and SIN recognition, which are most often affected after losing the ability to detect binaural cues. We hypothesized that patients with SSD would exhibit significant alterations in intrinsic brain activity in sensory- and cognitive-related brain regions. In addition, we conjectured that SSD patients with longer durations of deafness would exhibit better hearing abilities than those with shorter durations of deafness. Furthermore, we hypothesized that alterations in intrinsic brain activity may be closely related to hearing abilities in patients with SSD and act as compensatory mechanisms to facilitate improvement in hearing abilities over time.

## Materials and methods

### Subjects

A total of 57 subjects participated in this study, including 21 NHs (12 men, 41.3 ± 14.4 years old), 19 patients with left SSD (LSSD, 13 men, 44.1 ± 10.5 years old), and 17 patients with right SSD (RSSD, 7 men, 39.1 ± 9.4 years old). All subjects were native speakers of Mandarin and had no history of neurological or mental illness or contraindications to MRI scans. The demographic information for these subjects is presented in Table 1. There was no significant difference in age, sex, handedness, or education time among individuals in the three groups. All the durations of deafness were longer than 2 years in individuals in the SSD group, and the durations were not different between patients in the LSSD and RSSD groups. Among all patients with SSD, three with LSSD and two with RSSD could not provide a clear onset age of deafness and probably had prelingual onset. All other patients with SSD had postlingual onset. There was no significant difference in the age of deafness onset between participants in the two SSD groups. No history of hearing aid usage was reported by any patient with SSD.

**Table 1:** Demographic characteristics of the three groups.

	**LSSD** **(*n* = 19)**	**RSSD** **(*n* = 17)**	**NH** **(*n* = 21)**	**Statistics**	
Sex (male/female)	13/6	7/10	12/7	*χ^2^* = 2.72	*p* = 0.257
Age [year, mean (SD)]	44.1 (10.5)	39.1 (9.4)	41.3 (14.4)	*F* = 0.81	*p* = 0.451
Handedness (right/left)	18/1	15/2	20/1	Fisher's exact test = 0.97	*p* = 0.674
Education time [year, mean (SD)]	14.6 (3.4)	14.3 (4.2)	15.2 (2.9)	Kruskal–Wallis test = 0.61	*p* = 0.736
Duration of deafness [year, mean (SD)]	11.3 (11.2)	9.3 (12.2)	–	*t* = 0.52	*p* = 0.608
Age of deafness onset [year, mean (SD)]	32.7 (18.6)	29.8 (15.2)	–	*t* = 0.52	*p* = 0.606

### Audiological inclusion criteria

In our study, normal hearing was defined as air-conduction pure-tone audiometry (PTA) threshold of 25 dB HL or less from 0.5 to 2 kHz. The average PTA threshold was defined as the average air conduction threshold at 0.5, 1, 2, and 4 kHz. In the NH group, all subjects had normal hearing in their bilateral ears (Table 2). All patients with SSD had persistent severe to profound sensorineural hearing loss with an average PTA threshold of deaf ear >70 dB HL and had normal hearing on the other side (Table 2). The average PTA threshold was 98.82 ± 17.03 and 100.07 ± 17.49 dB HL in the LSSD and RSSD groups, respectively, and showed no significant difference (*t* = −0.22, *p* = 0.828) between them.

**Table 2:** Auditory abilities of the left single-sided deafness, right single-sided deafness, and normal hearing control groups.

	**LSSD** **(*n* = 19)**	**RSSD** **(*n* = 17)**	**NH** **(*n* = 21)**	**Statistics**			
				**ANOVA**	***Post-hoc*** **test**		
					**LSSD vs. NH**	**RSSD vs. NH**	**LSSD vs. RSSD**
Average PTA of normal ear [dB HL, mean (SD)]	16.32 (7.15)	14.78 (6.18)	12.79* (5.74)	*F* = 1.54, *p* = 0.223	*p* = 0.259	*p* = 1	*p* = 1
SIN threshold [dB, mean (SD)]	2.73 (1.59)	3.00 (1.68)	−6.88** (1.64)	*F* = 236.02, *p* <0.001	*p* <0.001	*p* <0.001	*p* = 1
ASL [%, mean (SD)]	35.32 (9.21)	32.13 (9.17)	84.95 (6.49)	*F* = 253.01, *p* <0.001	*p* <0.001	*p* <0.001	*p* = 0.759
RMS error [°, mean (SD)]	64.37 (17.05)	72.30 (18.01)	17.01 (4.19)	*F* = 88.19, *p* <0.001	*p* <0.001	*p* <0.001	*p* = 0.295

*The average PTA of normal ears in the NH group is the average value of both ears.

**The SIN threshold in the NH group is the average value of the SIN threshold for the left and right sides.

NH, normal hearing control; LSSD, left single-sided deafness; RSSD, right single-sided deafness; PTA, pure tone audiometry; SIN, speech-in-noise; ASL, accuracy rate of sound localization; RMS, root-mean-square. ANOVA, analysis of variance.

### Evaluation of higher-order hearing abilities

#### SIN recognition evaluation

The SIN recognition test was implemented using the Hearing-in-Noise Test (HINT, Version 7.2; Bio-logic Systems Corp, Mundelein, IL, USA), which was administered in a soundproof booth. The speech material was the Mandarin HINT (Wong et al., 2007). The SIN threshold on the deaf side was measured for patients in the two SSD groups. The sentence materials were presented by a speaker on the deaf side 1 m from the subjects, while noise was presented by a speaker in front of the participant. For participants in the NH group, we evaluated the SIN thresholds on the left side and right side (with noise presented in the front), and the average value of both sides was recorded as their SIN threshold. The speech-shaped noise masker was fixed at an intensity of 65 dB SPL. The speech signals were presented beginning at a −10 dB signal-to-noise ratio and adjusted according to the correct or wrong response provided by the subjects. The threshold was defined as the signal-to-noise ratio at which the subjects repeated sentences correctly 50% of the time.

#### Sound localization evaluation

Sound localization evaluation was carried out in the sound field of a soundproof booth. Thirteen loudspeakers (15° apart and numbered 1-13) were horizontally placed in a 180° arc in front of the subjects, with the subject as the center, with a radius of 1 m. The height of the sound field speakers was consistent with the height of the subject's ears. During the test, the subjects were instructed to remain still and face forward. Low-frequency (0.5 kHz) and high-frequency (3 kHz) pure tones at 50 dB HL were randomly presented two times from each of the 13 speakers as sound stimuli. After each sound stimulus, subjects were instructed to determine from which speaker the sound came and report the speaker number. When the deviation between the speaker location reported by the subject and the actual position of the stimulus was ≤ 15°, the answer was defined as correct. The correct rate was recorded as the accuracy of sound localization (ASL). The root-mean-square (RMS) error between the azimuth of the speaker location and the listener's response was also used to quantify localization accuracy. A higher ASL value indicated better sound localization ability, while a higher RMS error indicated greater deviation in identifying the sound source position, suggesting poorer sound localization capability.

### MRI acquisition

All MRI data were acquired on a 3 T Philips Achieva MRI scanner (Philips Healthcare, Best, The Netherlands) with a 32-channel head coil. Subjects were instructed to remain still in a supine position. Headphones and foam padding were used to reduce scanner noise and limit head motion. Subjects kept their eyes closed but remained awake during scanning. Resting-state functional images were collected axially using an echo-planar imaging (EPI) sequence with the following settings: 37 slices; slice thickness = 3.5 mm; gap = 0.5 mm; repetition time (TR) = 2,000 ms; echo time (TE) = 30 ms; flip angle (FA) = 90°; field of view (FOV) = 230 × 230 mm^2^; and sampling matrix = 80 × 80. The resting-state scan lasted 368 s (184 volumes). Three-dimensional T1-weighted magnetization-prepared rapid-acquisition gradient-echo (MPRAGE) coronal images were collected by using the following settings: slice thickness = 1.0 mm without gap; TR = 7.6 ms; TE = 3.7 ms; FA = 8°; FOV = 256 × 256 mm^2^; and sampling matrix = 256 × 256 × 180.

### fMRI preprocessing

Data preprocessing was performed with Data Processing & Analysis for (Resting-State) Brain Imaging (DPABI V5.1) (Yan et al., 2016) based on Statistical Parametric Mapping (SPM12, http://www.fil.ion.ucl.ac.uk/spm). The first 10 volumes of the acquired fMRI images for each subject were discarded for magnetization equilibrium and the subject's adaptation to scanning noise. Then, slice timing and motion correction were performed. All participants were retained under the head motion criteria of translation <2 mm or rotation <2° in any direction. The remaining fMRI time series was coregistered to the T1 images. Then, the T1 images were normalized to the Montreal Neurological Institute (MNI) space, and the resulting deformation fields were used to project the functional images to the MNI space with a voxel size of 3^*^3^*^3 mm. Nuisance covariate regression including Friston 24 parameters (Friston et al., 1996) was performed to remove the effects of head motion. In addition, the linear trend of time courses was removed. Then, the functional images were spatially smoothed with a 6-mm full width at a half-maximum Gaussian kernel.

### Calculation of ALFF values

The ALFF values of the preprocessed functional images were calculated using DPABI. Briefly, the time courses were first transformed to the frequency domain using the fast Fourier transform. The square root of the power spectrum obtained by fast Fourier transform was computed and then averaged across 0.01–0.08 Hz at each voxel, which was then taken as the ALFF value. To reduce the global effects of variability across the subjects and achieve standardization, the individual data were transformed to Z scores (i.e., the global mean value is subtracted from the score, and then the result is divided by the standard deviation) (Zou et al., 2008). Finally, we obtained the standardized whole-brain ALFF map.

### Statistical analysis

#### Demographic and auditory data

Statistical analysis of the demographic and auditory data was performed using the SPSS 23.0 statistical package (SPSS Inc., Chicago, IL, USA). The age differences among individuals in the three groups were tested by analysis of variance (ANOVA). Sex and handedness differences among individuals in the groups were analyzed by the chi-square test and Fisher's exact test, respectively. The differences in education time among individuals in the groups were analyzed by the Kruskal–Wallis test. The differences in age and auditory parameters among individuals in the three groups were tested by analysis of variance (ANOVA), and then *post-hoc* tests were conducted by Bonferroni correction. The intergroup difference in PTA thresholds of the deaf ear between patients with LSSD and RSSD was tested using a two-sample *t-*test.

To explore the effect of deafness time on higher-order hearing abilities, we took the median duration of deafness (3 years) as the time point and used a two-sample *t-*test to compare the difference in higher-order hearing abilities of SSD patients with durations of deafness <3 years (including 3 years) and those with durations of more than 3 years. Considering that the duration of deafness in SSD did not conform to a normal distribution, Spearman's rank correlation analysis was used to explore the correlation between the duration of deafness and higher-order hearing abilities.

#### ALFF analysis

An ALFF analysis was performed with the Resting-State fMRI Data Analysis Toolkit (REST 1.8, http://rest.restfmri.net). To explore the within-group ALFF pattern, one-sample *t-*tests were performed on the individual ALFF maps in a voxelwise way for each group. The within-group statistical threshold was set at *Z* > 3.09 (voxel-level *p* < 0.001 and cluster-level *p* < 0.05, one-tailed) (Wang et al., 2011). The Gaussian random-field theory (GRF) correction was used to correct multiple comparisons. This correction was confined within the gray matter mask obtained by selecting a threshold of 0.2 on the mean gray matter map of all subjects (volume = 53,156 voxels). To compare the differences in the ALFF pattern, voxelwise two-sample *t-*tests were performed on the ALFF map between NHs and patients with LSSD and between NHs and patients with RSSD. Participants' age and sex were controlled as covariates. The between-group statistical threshold was set at | *Z* | > 2.58 (voxel-level *p* < 0.01 and cluster-level *p* < 0.05, two-tailed). GRF correction was used for correcting multiple comparisons, and this correction was also confined within the group gray matter mask. To further observe the different trends of the ALFF values between groups, region-of-interest (ROI)-wise two-sample *t-*tests were performed. The ROI was defined as a sphere with a radius of 10 mm (containing 171 voxels) and centered at the peak point of clusters in each contrast.

#### Correlation analysis

To explore the relationship between the ALFF values and duration of deafness in the patients with SSD, voxelwise partial correlation analysis was performed between the ALFF values and duration of deafness in patients with LSSD and RSSD together, controlling for the effects of age and sex. To explore the relationship between the ALFF values and higher-order hearing abilities in patients with SSD, voxelwise partial correlation analysis was also performed between ALFF values and hearing abilities of patients with SSD, including SIN threshold, ASL, and RMS error, controlling for the effects of age and sex. The statistical threshold was set at | *Z* | > 1.96 (voxel-level *p* < 0.05 and cluster-level *p* < 0.05, two-tailed) with GRF correction (Wang et al., 2011). Through the above voxelwise partial correlation analysis, brain areas showing a significant correlation between ALFF values and clinical parameters were found. We also performed ROI-wise partial correlation analysis, controlling for the effects of age and sex, between higher-order hearing abilities and the averaged ALFF values of the abovementioned areas.

#### Mediation analysis

Mediation analysis was performed using model 4 (simple mediation model) of the PROCESS (v3.3) macro in SPSS (Hayes and Ph, 2012). This model used a non-parametric bootstrap test with 5,000 resamplings to calculate the 95% confidence intervals for statistical significance. The mediation effect of the ALFF value on the relationship between deafness duration and higher-order hearing abilities was tested by controlling for sex and age (more details are provided in the Supplementary materials).

## Results

### Demographic characteristics and auditory abilities

As presented in Table 1, there were no differences among NHs, LSSD patients, and RSSD patients in sex (χ^2^ = 2.72, *p* = 0.257), age (*F* = 0.81, *p* = 0.451), handedness (*Fisher's exact test* = 0.97, *p* = 0.674), or education time (*Kruskal—Wallis test* = 0.61, *p* = 0.736). The duration of deafness (*t* = 0.52, *p* = 0.608) and the age of deafness onset (*t* = 0.52, *p* = 0.606) were not significantly different between patients with LSSD and RSSD.

The results of auditory ability are presented in Table 2. The average PTA of normal ears was not significantly different among NHs, patients with LSSD, and patients with RSSD (*F* = 1.54, *p* = 0.223). For the SIN recognition evaluation, the SIN threshold of NHs was significantly lower than that of patients with LSSD and RSSD (*F* = 236.02, *p* < 0.001), suggesting better performance in NHs. In the sound localization evaluation, NHs showed significantly higher ASL than did patients with LSSD or RSSD (*F* = 253.01, *p* < 0.001) and significantly lower RMS error than patients with LSSD or RSSD (*F* = 88.19, *p* < 0.001). Both results suggest better sound localization abilities in NHs than in patients with SSD, whether left or right. There was no difference between patients with LSSD and RSSD in the average PTA of the normal ear, SIN threshold, ASL, or RMS error.

**Figure 1:**
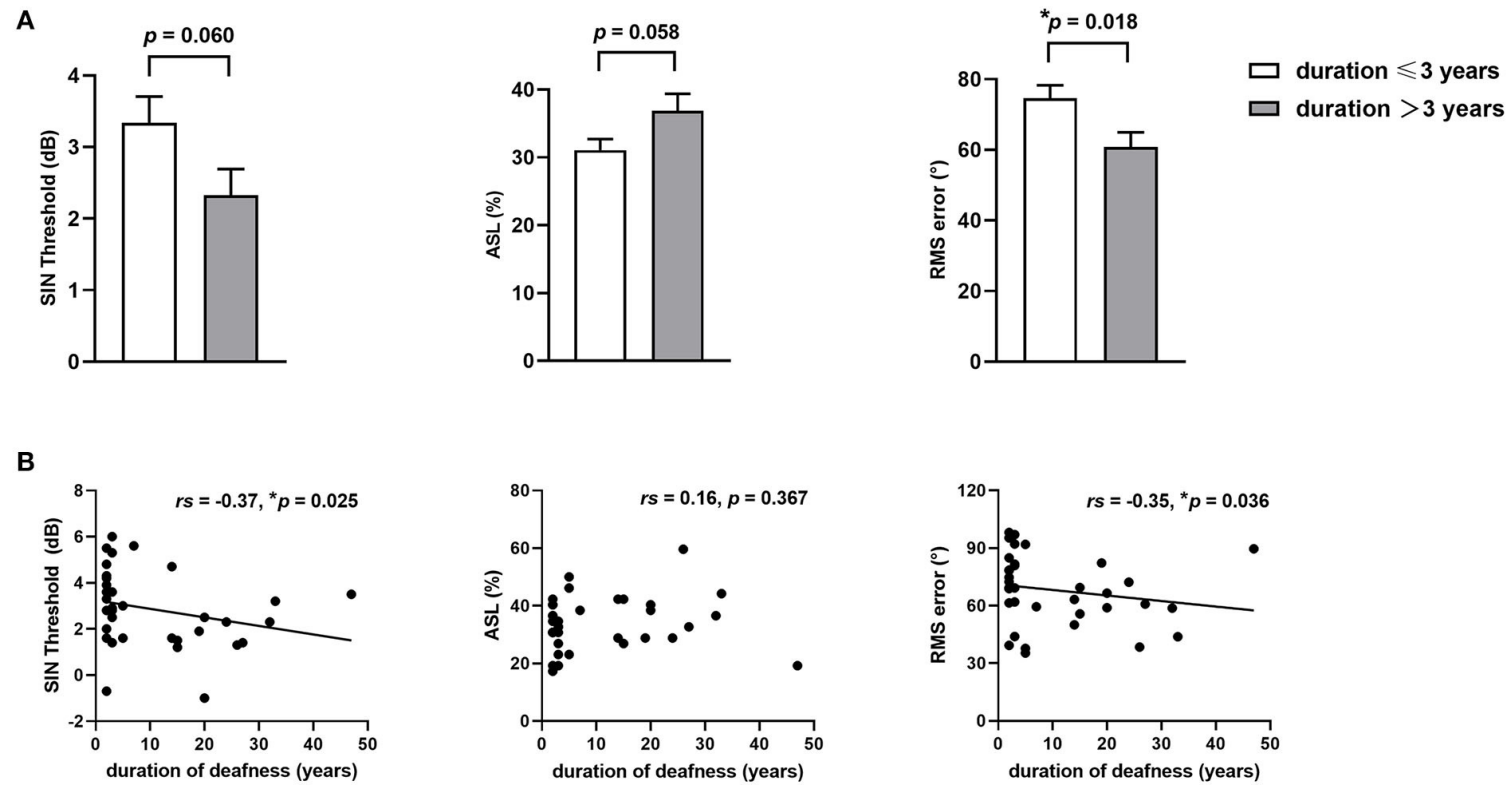
Higher-order hearing abilities in patients with SSD with different durations of deafness. **(A)** Comparison of higher-order hearing abilities, including the SIN threshold, ASL, and RMS error, between SSD patients with durations of deafness of up to 3 years and more than 3 years. **(B)** Spearman' correlations between duration of deafness and higher-order hearing abilities, including the SIN threshold, ASL, and RMS error. *p < 0.05, SSD, single-sided deafness; SIN, speech-in-noise; ASL, accuracy rate of sound localization; RMS, root-mean-square.

The results of higher-order hearing abilities in SSD patients with different durations of deafness are shown in Figure 1. Taking the median duration of deafness (3 years) as the time point, we compared the higher-order hearing abilities of SSD patients with deafness durations <3 years (including 3 years) and longer than 3 years. There was no significant age difference between participants in the two groups (*t* = 0.14, *p* = 0.257). Although the SSD patients with deafness durations <3 years had lower average PTA both for deaf ears (*t* = −3.25, *p* = 0.002) and for normal ears (*t* = −2.03, *p* = 0.048) than SSD patients with longer deafness durations, SSD patients with longer deafness durations showed a significant reduction in RMS error (*t* = −2.49, *p* = 0.018), a marginally significant reduction in the SIN threshold (*t* = −1.95, *p* = 0.060), and a marginally significant increase in ASL (*t* = 1.97, *p* = 0.057) than SSD patients with deafness durations of <3 years (see Figure 1A). The results of Spearman's correlation analysis between the duration of deafness and higher-order hearing abilities are shown in Figure 1B. The duration of deafness showed a significant negative correlation with the SIN threshold (*rs* = −0.37, *p* = 0.025) and RMS error (*rs* = −0.35, *p* = 0.036), indicating that duration was positively correlated with hearing abilities. However, there was no significant correlation between ASL and duration of deafness (*rs* = 0.16, *p* = 0.367).

### ALFF results

**Figure 2:**
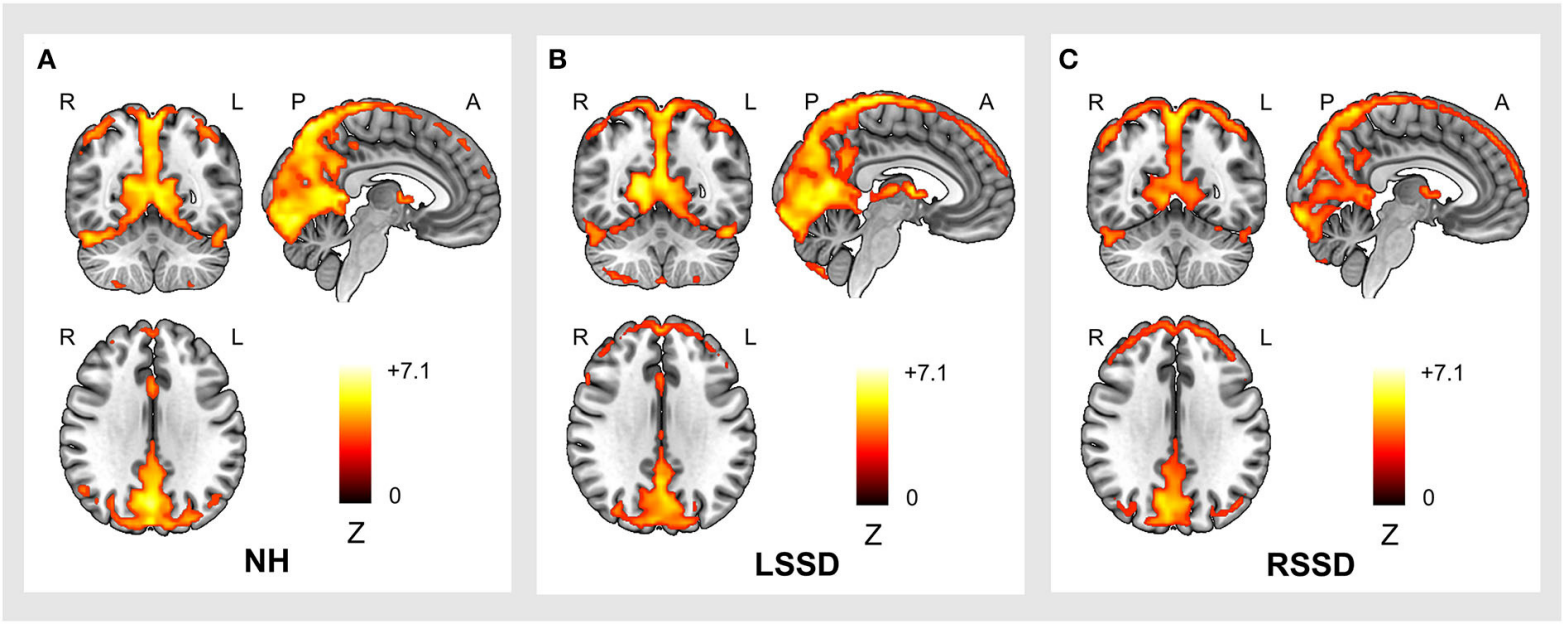
Within-group ALFF patterns of participants in the NH **(A)**, LSSD **(B)**, and RSSD **(C)** groups. NH, normal hearing control; LSSD, left single-sided deafness; RSSD, right single-sided deafness.

The within-group ALFF patterns of NHs, patients with LSSD, and patients with RSSD are shown in Figure 2. Visually, participants in all three groups showed similar patterns with higher ALFF values in the PCUN, IPL, posterior cingulate gyrus (PCG), medial prefrontal cortex (MPFC), and occipital areas. From the color intensity of Figure 2, it can be observed that participants in the NH group showed generally higher ALFF values than participants in the LSSD and RSSD groups.

**Figure 3:**
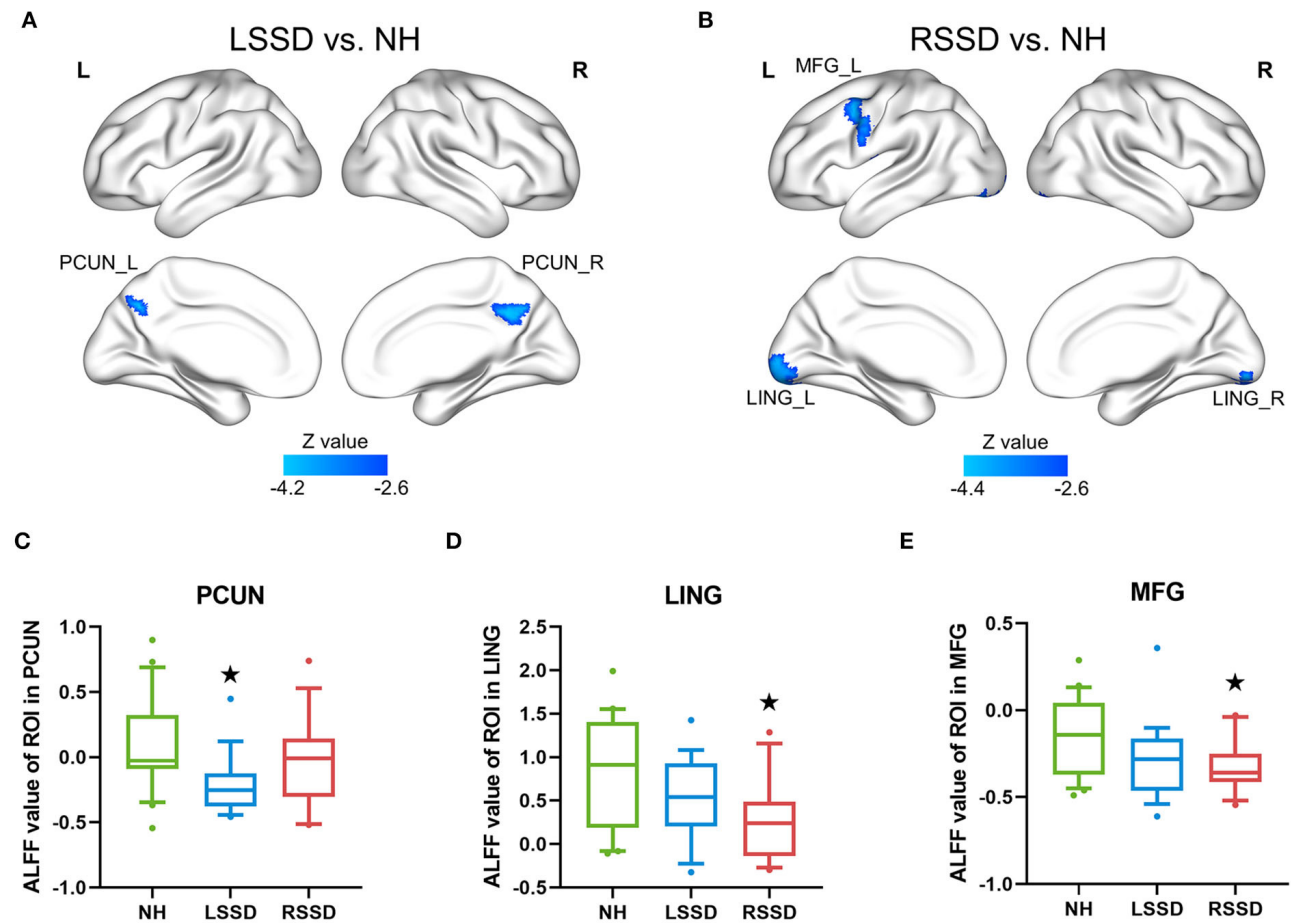
Differences in ALFF values among groups. **(A)** The patients with LSSD showed significantly lower ALFF values than NHs in the bilateral PCUN in the voxelwise comparison. **(B)** The patients with RSSD showed significantly lower ALFF values than NHs in the bilateral LING and left MFG in the voxelwise comparison. **(C)** Box plots showing the ALFF values of the three groups in the PCUN ROI. **(D)** Box plots showing the ALFF values of the three groups in the LING ROI. **(E)** Box plots showing the ALFF values of the three groups in the MFG ROI. The centerline indicates the median, box outlines show the 25th and 75th percentiles, and whiskers indicate the 10th–90th percentile. Extreme values are shown by dots. ^⋆^*p* < 0.05/3 = 0.017 (Bonferroni corrected) compared with those of NHs. NH, normal hearing control; LSSD, left single-sided deafness; RSSD, right single-sided deafness; PCUN, precuneus; LING, lingual gyrus; MFG, middle frontal gyrus.

The results of the between-group ALFF analysis are shown in Figure 3 and Table 3. The voxelwise between-group analysis showed that patients with LSSD exhibited significantly lower ALFF values in the bilateral PCUN than NHs (peak MNI = 12, −51, 36; *Z* = −4.13; cluster size = 81 voxels) (see Figure 3A). The patients with RSSD showed lower ALFF values than NHs in the bilateral lingual gyrus (LING, peak MNI = −18, −90, −9; *Z* = −4.34; cluster size = 149 voxels) and the left middle frontal gyrus (MFG, peak MNI = −36, 6, 48; *Z* = −4.06; cluster size = 102 voxels) (see Figure 3B). To further explore whether the patients with RSSD and patients with LSSD exhibited a similar trend of alteration, we performed an ROI analysis using the peak points found above as the center. For the PCUN ROI, obtained from the peak point of voxelwise analysis between patients with LSSD and NHs, patients with LSSD exhibited significantly lower ALFF values than NHs (*t* = 3.26, *p* = 0.002), and patients with RSSD exhibited lower ALFF values than NHs by a statistically nonsignificant margin (*t* = 0.99, *p* = 0.328) (see Figure 3C). Patients with RSSD exhibited significantly lower ALFF values than NHs in the ROIs of the LING (*t* = 2.91, *p* = 0.006) and MFG (*t* = 2.66, *p* = 0.012), and patients with LSSD showed lower ALFF values in the ROIs of the LING (*t* = 1.60, *p* = 0.118) and MFG (*t* = 1.78, *p* = 0.084) but without statistical significance (see Figures 3D,E).

**Table 3:** Brain regions showing significant between-group differences in ALFF values.

**Contrast**	**Region**	**Brodmann's area**	**Maximum Z value**	**Cluster size**	**MNI coordinates**		
					**X**	**Y**	**Z**
**NH vs. LSSD**							
	Bilateral precuneus	7/23	−4.13	81	12	−51	36
**NH vs. RSSD**							
	Bilateral lingual gyrus	17/18	−4.34	149	−18	−90	−9
	Left middle frontal gyrus	6	−4.06	102	−36	6	48

NH, normal hearing control; LSSD, left single-sided deafness; RSSD, right single-sided deafness.

### Correlation results

**Figure 4:**
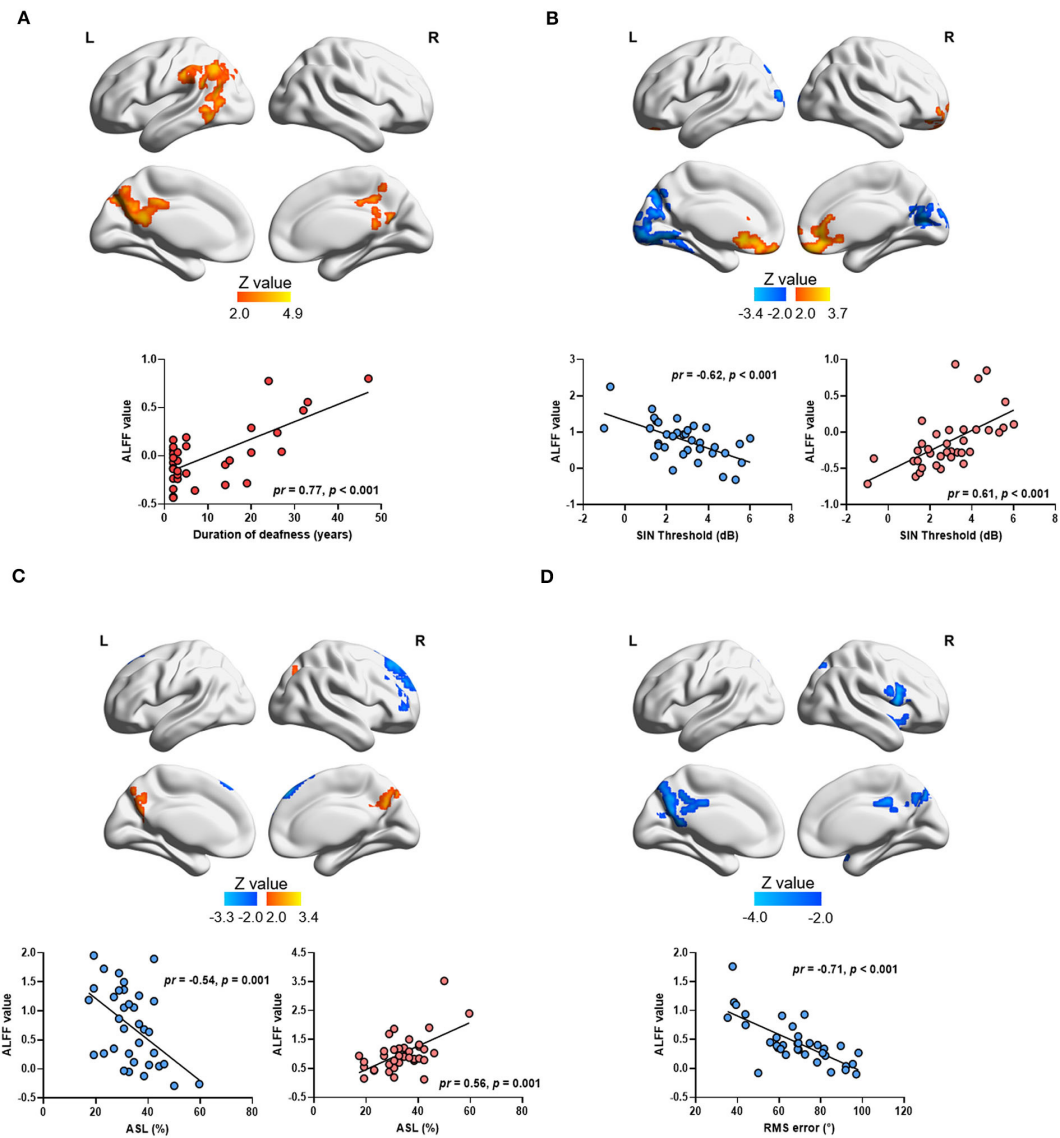
Correlation between ALFF values and auditory parameters in single-sided deafness after controlling sex and age. The brain map in the top panel shows the results of voxelwise correlation, and the scatterplot in the bottom panel shows the ROI-wise correlation between ALFF values and auditory parameters. The ALFF values in ROI-wise correlation analysis were extracted from the significant brain area in the voxelwise correlation in the top panel. Brain regions with positive and negative correlations were extracted separately. **(A)** Regions showing a significant correlation between the duration of deafness and ALFF values. **(B)** Regions showing a significant correlation between ALFF values and the SIN threshold. **(C)** Regions showing a significant correlation between ALFF values and ASL. **(D)** Regions showing a significant correlation between ALFF values and RMS error. SIN, speech-in-noise; ASL, accuracy rate of sound localization; RMS, root-mean-square.

A voxelwise correlation map between ALFF values and the duration of deafness is shown in Figure 4. A significantly positive correlation was shown between the duration of deafness and ALFF values in the left IPL, the left angular gyrus (ANG), the left middle occipital gyrus (MOG), and the bilateral PCUN and extending to the PCG (see Figure 4A). The scatterplot of ROI-wise analysis displayed a trend of a significant positive correlation (*pr* = 0.77, *p* < 0.001) between ALFF values and duration of deafness (see Figure 4A).

Correlations between ALFF values and higher-order hearing abilities in all SSD subjects are also shown in Figure 4. A significant negative correlation was observed between the SIN thresholds and ALFF values in the left superior occipital gyrus (SOG), the left LING, bilateral calcarine (CAL), and the bilateral PCUN (see Figure 4B and Table 4). At the same time, a significantly positive correlation was observed between the SIN threshold and ALFF values in the bilateral MFG and anterior cingulate gyrus (ACG) (see Figure 4B and Table 4). The scatterplot of ROI-wise analysis is displayed in the bottom panel for ROIs extracted from regions showing significant negative correlations (*pr* = −0.62, *p* < 0.001) and positive correlations (*pr* = 0.61, *p* < 0.001) between SIN thresholds and ALFF values (see Figure 4B). A significant negative correlation was observed in the right SFG and right MFG, and a significant positive correlation was observed in the bilateral PCUN between ALFF values and ASL (see Figure 4C and Table 4). The scatterplot for ROIs extracted from regions showing a significant negative correlation (*pr* = −0.54, *p* = 0.001) and positive correlation (*pr* = 0.56, *p* = 0.001) between ASL and ALFF values is displayed (see Figure 4C). A significant negative correlation was revealed between RMS error and ALFF values in the right IFG, bilateral PCUN, and bilateral PCG (see Figure 4D and Table 4). The scatterplot for the ROIs extracted from regions showing a significant negative correlation (*pr* = −0.71, *p* < 0.001) between RMS error and ALFF values is displayed in the bottom panel.

**Table 4:** Brain regions showing significant correlations between ALFF values and auditory parameters in voxelwise correlation analysis in SSD.

**Auditory parameters**	**Region**	**Brodmann's area**	**Maximum *Z*-value**	**Cluster size**	**MNI coordinates**		
					**X**	**Y**	**Z**
**Duration of deafness**							
**Positive correlation**							
	Left inferior parietal lobule	40	4.92	189	−51	−39	36
	Left angular gyrus	39	4.679	110	−45	−60	39
	Left middle occipital gyrus	19		97			
	Bilateral precuneus	7/23	4.34	254	−18	−63	33
	Bilateral posterior cingulate	30	3.41	81	−3	−48	21
**SIN threshold**							
**Positive correlation**							
	Bilateral medial frontal gyrus	11	3.72	134	15	24	−6
	Bilateral anterior cingulate			106			
**Negative correlation**							
	Left superior occipital gyrus	19	−3.40	47	−18	−84	42
	Left precuneus	7		81			
	Left lingual gyrus	18	−3.20	146	−12	−63	−6
	Left calcarine	17		52			
	Right calcarine	17/18	−3.09	111	18	−72	15
	Right precuneus	23		112			
**ASL**							
**Positive correlation**							
	Bilateral precuneus	7	3.40	187	3	−72	45
**Negative correlation**							
	Right superior frontal gyrus	8/9/10	−3.33	264	27	66	9
	Right middle frontal gyrus			152			
**RMS error**							
**Negative correlation**							
	Right inferior frontal gyrus	44/6	−4.00	64	51	9	15
	Bilateral precuneus	7	−4.01	224	−3	−60	33
	Bilateral cingulate gyrus	23		97			

SIN, speech-in-noise; ASL, accuracy rate of sound localization; RMS, root-mean-square.

### Mediation analysis results

**Figure 5:**
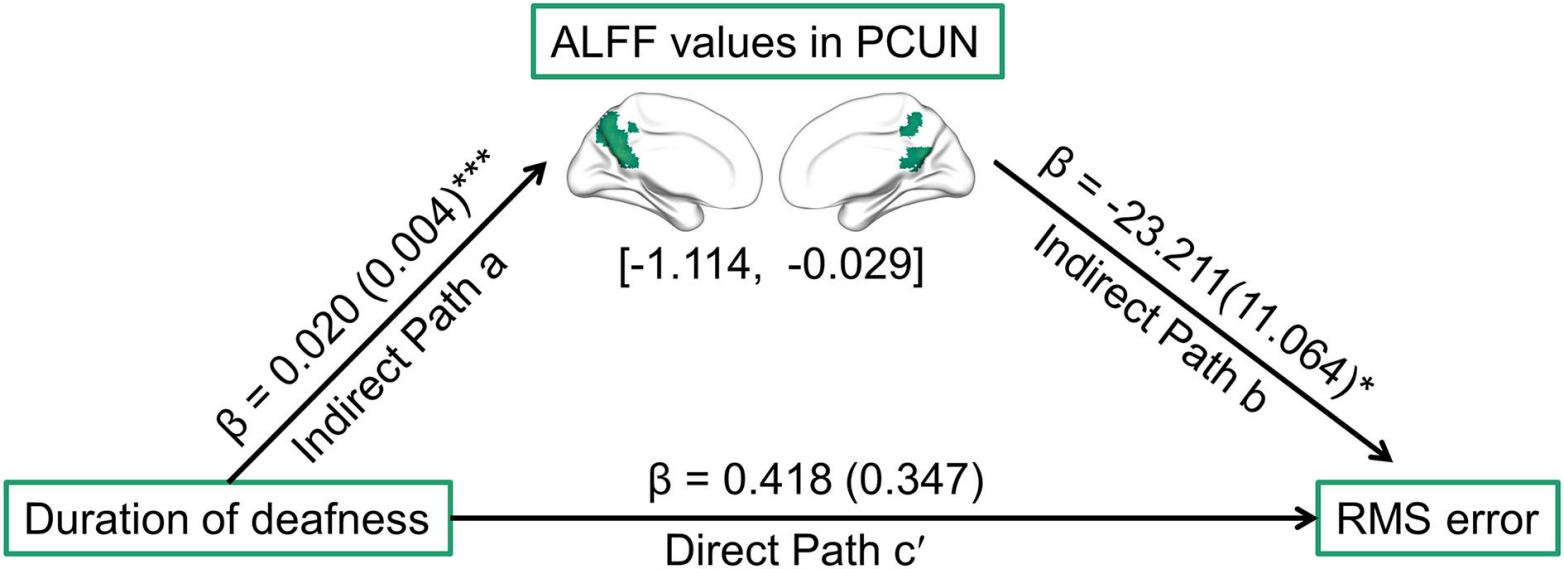
Path diagram showing the relationships among duration of deafness, ALFF values in ROIs located in the PCUN, and RMS error in patients with SSD according to mediation analysis. The lines are labeled with path coefficients, and standard errors are shown in parentheses. The values in brackets are the upper and lower limits of the bootstrap 95% confidence interval for indirect effects. The predictor (duration of deafness) connection to the mediator factors (ALFF values in the PCUN) is indirect path a. The connection from the mediator factor (ALFF values in the PCUN) to the outcome (RMS error) is indirect path b. The connection from the predictor (duration of deafness) to the outcome (RMS error) is direct path c′. *p < 0.05, ***p < 0.001. RMS, root-mean-square; PCUN, precuneus.

As described above, a significant correlation was observed in patients with SSD between the duration of deafness and higher-order abilities, and ALFF values in the PCUN were observed to be correlated with both these aspects. Thus, it was speculated that ALFF values in the PCUN may be a mediator of the relationship between the duration of deafness and higher-order hearing abilities, including SIN threshold, ASL, and RMS error. The ALFF values were extracted in the ROIs located in the PCUN, which were defined by the overlap between the PCUN, as delineated by the Automated Anatomical Labeling atlas, and regions showing a significant correlation between duration of deafness and ALFF values. The results of the mediation analysis are shown in Figure 5. ALFF values in the PCUN had a significant negative predictive effect on RMS error (β = −23.211, SE = 11.064, *p* = 0.044). Furthermore, the indirect effect of duration of deafness on RMS error was significant [95% CI = (−1.114, −0.029)], while duration of deafness had no significant direct predictive effect on RMS error in the mediation model (β = 0.418, SE = 0.347, *p* = 0.237). Therefore, ALFF values in the PCUN are a significant mediator of the relationship between the duration of deafness and RMS error. However, ALFF values in the PCUN showed no significant mediating effect in the relationship between duration of deafness and SIN threshold [95% CI = (−0.057, 0.036)] or in the relationship between duration of deafness and ASL [95% CI = (0.000, 0.006)] (more details are provided in the Supplementary materials).

## Discussion

In the present study, we investigated the alteration in intrinsic brain activities and their correlations with higher-order abilities in patients with long-term SSD using ALFF of resting-state fMRI. Our study provided several key findings. First, we confirmed that SSD patients with longer durations of deafness had better higher-order hearing abilities. Second, we observed a consistent trend of decreased ALFF values in multiple brain areas for both patients with LSSD and patients with RSSD. Third, higher ALFF values were observed to correlate with longer durations of deafness in multiple parietal-occipital regions, especially the PCUN. Furthermore, a generally consistent trend of correlation between ALFF values in specific brain areas and higher-order hearing abilities was observed in patients with SSD. That is, better abilities correlated significantly with lower ALFF values in the frontal areas and higher ALFF values in the PCUN and the surrounding parietal-occipital regions for both SIN recognition and sound localization. Finally, mediation analysis revealed that ALFF values in the PCUN were a significant mediator of the relationship between the duration of deafness and higher-order hearing abilities.

Due to hearing deprivation in one ear, no binaural cues (e.g., interaural time difference, intensity difference, and binaural squelch) could be detected by the peripheral auditory system in patients with SSD. Since these cues are crucial for sound localization and SIN recognition, these hearing abilities are most affected in patients with SSD (Agterberg et al., 2014; Asp et al., 2018; Liu et al., 2018; Adigun and Vangerwua, 2021). However, according to our behavioral results, SSD patients with longer durations of deafness showed better sound localization ability, although their PTA thresholds were even higher than those of patients with shorter durations of deafness. Furthermore, a significant correlation between duration and hearing ability was observed for both SIN recognition and sound localization. These findings were consistent with previous behavioral studies in both children and adults (Peckham and Sheridan, 1976; Lieu et al., 2012; Liu et al., 2018; Nelson et al., 2018). In addition, studies have reported that sound localization may be improved by active training in patients with SSD (Firszt et al., 2015; Yu et al., 2018). These findings demonstrated that higher-order hearing abilities could be improved over time without the recovery of binaural cues. Researchers generally believe that, on the one hand, this outcome may be due to the adaptation to the loss of binaural cues over time *via* the remediation of other sound cues (Liu et al., 2018). On the other hand, central plasticity in patients with SSD may be an important mechanism that recruits more brain resources for auditory processing to make better usage of limited auditory input (Chang et al., 2016; Li et al., 2019).

Individuals in all three groups (NH, LSSD, and RSSD) showed higher ALFF values in brain regions of the default-mode network (DMN), including the PCUN, IPL, PCG, and MPFC, as well as occipital areas, which were consistent with previous studies of ALFF (Yan et al., 2009; Spunt et al., 2015; Mak et al., 2017; Jenkins, 2019). Studies have indicated that regions of the DMN in the human brain have a distinctive functional profile, with higher activity than other regions of the brain at baseline (Zang et al., 2007; Yan et al., 2009; Wang et al., 2011; Spunt et al., 2015; Mak et al., 2017; Jenkins, 2019; Jiang et al., 2020). Moreover, we observed a decreasing trend of ALFF in several regions in patients with SSD compared with those with NHs. Patients with LSSD showed significantly decreased ALFF in the bilateral PCUN. In many studies, ALFF on resting-state fMRI has been considered a promising neurophysiological marker reflecting intrinsic brain activity (Wang et al., 2011; Liu et al., 2014; Cheng et al., 2020). Pertinently, decreased ALFF may indicate brain dysfunction (Wang et al., 2011; Liu et al., 2014; Mu et al., 2020). The PCUN is considered a key functional hub in the DMN at rest and plays a distinct role in many high-level functions, such as episodic memory retrieval (Dörfel et al., 2009), self-processing (Lou et al., 2004), visuospatial processing (Wenderoth et al., 2005), and deductive reasoning (Knauff et al., 2003; see also Cavanna and Trimble, 2006 for review). An increasing body of evidence suggests that the PCUN participates in attentional monitoring and is responsible for continuously collecting and automatically distributing information from the self and the surrounding environment (Hutchinson et al., 2009; Halbertsma et al., 2020; Li et al., 2020). Consistent with the findings of the present study, Yang et al. observed decreased ALFF in the PCUN in patients with unilateral hearing loss (Yang et al., 2014). Studies have also observed altered functional connectivity of the DMN, including the PCUN, during the resting state (Wang et al., 2014; Zhang et al., 2015, 2018a; Shang et al., 2020) and have reported altered activation during tasks in DMN regions in patients with SSD (Schmithorst et al., 2014; Shang et al., 2018). Based on the information mentioned above, the decreased ALFF values in the PCUN observed in the present study may indicate an abnormality in higher-order cognitive function in patients with SSD after losing auditory input from one ear.

In the present study, we observed a significant positive correlation between deafness duration and ALFF values in the bilateral PCUN and the surrounding parietal regions in patients with SSD. In other words, the longer the duration of deafness, the closer to normal the ALFF. This finding suggested a compensatory mechanism, that is, brain function tended to recover to a near normal state over time. Furthermore, we investigated the relationship between ALFF values and higher-order auditory function. For both SIN recognition and sound localization, better abilities correlated significantly with higher ALFF values in the bilateral PCUN. Together with our behavioral findings that patients with longer durations showed better auditory performance, it could be conjectured that the recovery of ALFF values in the PCUN may be one of the mechanisms mediating the compensation of higher-order auditory function. The results of the mediation analysis revealed that ALFF values in the PCUN showed a significant mediation effect on the relationship between the duration of deafness and sound localization ability, which further confirmed this conjecture.

In addition to the PCUN, the MFG showed a significantly lower ALFF value in patients with SSD. Furthermore, a similar pattern was observed in the correlation analysis for both SIN recognition and sound localization, and better abilities were observed to be correlated with lower ALFF values in the frontal areas, including the SFG and MFG. The MFG is one of the secondary language areas that is involved in the nuances of language expression, such as grammar (Wang et al., 2008), semantics (Brown et al., 2006), and verbal fluency (Abrahams et al., 2003). There is also evidence suggesting that the MFG is involved in information storage and cognitive processing in working memory (Leung et al., 2002). The SFG has also been demonstrated to contribute to higher cognitive functions, particularly to working memory (du Boisgueheneuc et al., 2006; Alagapan et al., 2018). These results suggested that functional reorganization of intrinsic activity in the frontal lobe, particularly regions subserving working memory, not only occurred in patients with SSD but also had a close relationship with higher-order auditory abilities.

Previous studies have demonstrated that degraded peripheral input leads to increased processing demands, that is, listening effort, including increases in the attentional focus and time needed to process auditory information (Shinn-Cunningham and Best, 2008). In addition, more cognitive areas are engaged in auditory processing when more listening effort is required (Davis and Johnsrude, 2003; Tyler et al., 2010; Peelle et al., 2011; Hervais-Adelman et al., 2012; Peelle, 2018; Rosemann and Thiel, 2018). In NHs, both the ANG and the extensive prefrontal cortex were demonstrated to be recruited when higher-order linguistic factors improved speech comprehension under adverse listening conditions (Obleser et al., 2007). In adults with mild to moderate hearing loss, Campbell and Sharma observed increased activation in the frontal areas (e.g., the SFG, MFG, and IFG) when individuals tried to recognize speech when background noise was presented simultaneously (Campbell and Sharma, 2013), and Rosemann and Thiel observed higher activation in the medial, middle, and inferior frontal gyri during a task of incongruent audio-visual conditions that required more listening effort (Rosemann and Thiel, 2018).

The subjects with SSD have also been demonstrated to require more listening effort than NHs when performing the same auditory processing tasks (Lewis et al., 2016). Previous data-driven studies in SSD have demonstrated that both structural and functional reorganization in cognitive-related regions and networks are the most important patterns of plasticity (Zhang et al., 2018a,b; Li et al., 2019; Zhu et al., 2021). Furthermore, Li et al. observed a strong correlation between hearing abilities and connection strength, mainly in the frontoparietal areas (Li et al., 2019). A previous auditory working memory task study in patients with SSD using magnetoencephalography observed reduced gamma band activity over the frontoparietal cortices related to attention and working memory, and the author conjectured that the attention and working memory network were overburdened chronically in patients with SSD such that no comparable resources could be allocated relative to the resources available to NHs while performing challenging auditory tasks (Shang et al., 2018). Our results further demonstrated that the functional reorganization of the DMN and other cognitive-related regions, especially those subserving attention and working memory, contribute to the compensatory mechanism for the recovery of hearing abilities in patients with SSD. These alterations happen not only during auditory processing but also in intrinsic brain activity during the resting state.

In the current study, significantly decreased ALFF values were observed in the bilateral LING in patients with RSSD, and there was a similar lower alteration trend in patients with LSSD, but the difference was not statistically significant. Furthermore, brain regions showing significant correlations between ALFF values and deafness durations involved the left MOG; moreover, brain regions showing significant correlations between ALFF values and SIN recognition involved the left SOG. These findings suggest that the intrinsic activity of the visual cortex was reorganized in patients with SSD and that this reorganization has a close relationship with auditory function, implying cross-modal plasticity. Cross-modal plasticity has been well-demonstrated in patients with bilateral severe to profound hearing loss, that is, total hearing deprivation. Recently, growing evidence has suggested that there is cross-modal plasticity in patients with partial hearing deprivation, that is, SSD. Structurally, decreased gray matter volume and decreased white matter structural network strength in visual brain regions were found in patients with SSD (Wang et al., 2016; Li et al., 2019). Functionally, altered regional homogeneity and functional connections in visual areas were also observed in patients with SSD using resting-state fMRI (Wang et al., 2014; Liu et al., 2015; Xu et al., 2016; Zhang et al., 2016, 2018a). Altered activation in the visual cortex was also observed in studies in which individuals performed audio-visual, visual, or auditory tasks (Propst et al., 2010; Schmithorst et al., 2014; Shang et al., 2018; Qiao et al., 2019). Our findings were consistent with those of these studies to some extent and further suggested that the functional reorganization of the visual cortex correlated closely with the recovery of auditory function.

Since quite a few previous studies on patients with SSD have reported significant alterations in the interhemispheric symmetry and synchronization of the auditory cortex, alterations in the ALFF values were expected in the auditory cortex (Ponton et al., 2001; Khosla et al., 2003; Langers et al., 2005). However, it is notable that the auditory cortex is not among the areas showing significant ALFF alterations or areas showing a close relationship between ALFF values and higher-order auditory functions. This is probably because, although the auditory input is abolished in the deaf ear, most of the auditory function is retained due to the normal input from the good ear. Thus, the basic function of the auditory cortex, especially the primary auditory cortex, remains unchanged. Using a data-driven approach, our results suggested that the intrinsic activity of the auditory cortex remains stable in patients with SSD; at least, it is not among the most obvious alterations. Similar findings were observed in other data-driven studies in patients with SSD SSD. A previous study of structural connectivity networks in patients with SSD observed increased connectivity strengths in the frontoparietal subnetwork and decreased connectivity strengths in the visual network but not in the auditory network (Li et al., 2019). A data-driven functional connectivity study in patients with SSD observed that brain regions showing the most obvious alterations are mainly those related to higher-order cognitive functions instead of the auditory cortex (Zhu et al., 2021). Another possible reason for this phenomenon is that the auditory cortex is not among the regions showing high ALFF values during the resting state. Thus, this region is less likely to exhibit reduced ALFF.

It has been well-accepted that there are two streams for auditory processing: a ventral “what” stream and a dorsal “where” stream (Hickok and Poeppel, 2000, 2004; Rauschecker and Tian, 2000). The dorsal stream is also involved in mapping sound to articulatory-based representations (Hickok and Poeppel, 2004; Elmer et al., 2017). In the present study, regions showing a close relationship between ALFF and duration of deafness involved the IPL and ANG, which are important parts of the dorsal processing pathway and are linked to the “phonological-articulatory loop” (Rauschecker and Scott, 2009). The ANG was demonstrated to be recruited when higher-order linguistic factors improve speech comprehension (Obleser et al., 2007). Our findings suggested that functional reorganization occurred in the dorsal auditory processing pathway over time, especially in regions related to higher-order linguistic functions. Furthermore, although SIN recognition and sound localization were believed to be processed by different mechanisms, a similar pattern was observed in voxelwise correlation analysis between ALFF and auditory abilities.

There were several limitations in the present study. First, the sample sizes for both the SSD and NH groups were relatively modest, resulting in reduced sensitivity for ALFF comparisons between groups. Voxelwise correlation analysis was implemented for all SSD subjects without differentiating the deafness laterality to achieve suitable statistical power. Second, the present study was not able to analyze the prelingual and postlingual SSD separately due to the relatively small size of prelingual patients in our cohort. Since there is a critical period for auditory development and plasticity pattern may be different between prelingual and postlingual SSD cases (Kral et al., 2013), further studies are still needed to clarify it. At last, the effects of other otological symptoms, such as tinnitus and vertigo, were not assessed. Brain function during the resting state has been demonstrated to be affected by tinnitus in previous imaging studies (Schmidt et al., 2013; Hinkley et al., 2015).

## Conclusion

In the present study, significant alterations in intrinsic brain activity were observed in multiple regions of the brain in patients with SSD, including cognitive-related regions. These alterations were closely related to the duration of deafness and higher-order hearing abilities. These findings suggested that alterations in intrinsic brain activity, especially in cognitive-related regions, may be one of the compensatory mechanisms that develop over the duration of deafness to restore the higher-order hearing abilities in patients with SSD.

## Data availability statement

The original contributions presented in the study are included in the article/Supplementary material, further inquiries can be directed to the corresponding author/s.

## Ethics statement

The studies involving human participants were reviewed and approved by Ethics Committee of Peking Union Medical College Hospital. The patients/participants provided their written informed consent to participate in this study.

## Author contributions

YQ and YSh contributed to the conception and design of the study. YQ, MZ, and WS contributed to data acquisition and organized the database. YQ, MZ, WS, and YSu performed the statistical analysis. YQ, MZ, HG, and YSh wrote the manuscript. All authors contributed to manuscript revision and read and approved the submitted version.

## Funding

The study was supported by the National Key Research and Development Program of China (Grant No. 2020YFC2005200) and the National Natural Science Foundation of China (Grant No. 82171156).

## Conflict of interest

The authors declare that the research was conducted in the absence of any commercial or financial relationships that could be construed as a potential conflict of interest.

## Publisher's note

All claims expressed in this article are solely those of the authors and do not necessarily represent those of their affiliated organizations, or those of the publisher, the editors and the reviewers. Any product that may be evaluated in this article, or claim that may be made by its manufacturer, is not guaranteed or endorsed by the publisher.

## Abbreviations

SSD: single-sided deafness
ALFF: amplitude of low-frequency fluctuation
NH: normal hearing control
LSSD: left single-sided deafness
RSSD: right single-sided deafness
PTA: pure-tone audiometry
SIN: speech-in-noise
ASL: accuracy rate of sound localization
RMS: root-mean-square.
