# Supplementary material for: Intrinsic brain activity reorganization contributes to long-term compensation of higher-order hearing abilities in single-sided deafness
Source: Front Neurosci. 2022 Aug 25;16:935834. doi: 10.3389/fnins.2022.935834 (PMC9453152; doi:10.3389/fnins.2022.935834)
Supplement: Supplementary file 1 [file Data_Sheet_1.docx]

Supplementary Material

**Mediation models and hypotheses**

The ALFF values of PCUN was the mediator for the relationship between duration of deafness and higher-order hearing abilities including SIN threshold, ASL and RMS error, respectively. The ALFF values were extracted in the ROIs located in PCUN (155 voxel), which were defined by the overlap of map with significant correlation between duration of deafness and ALFF values.


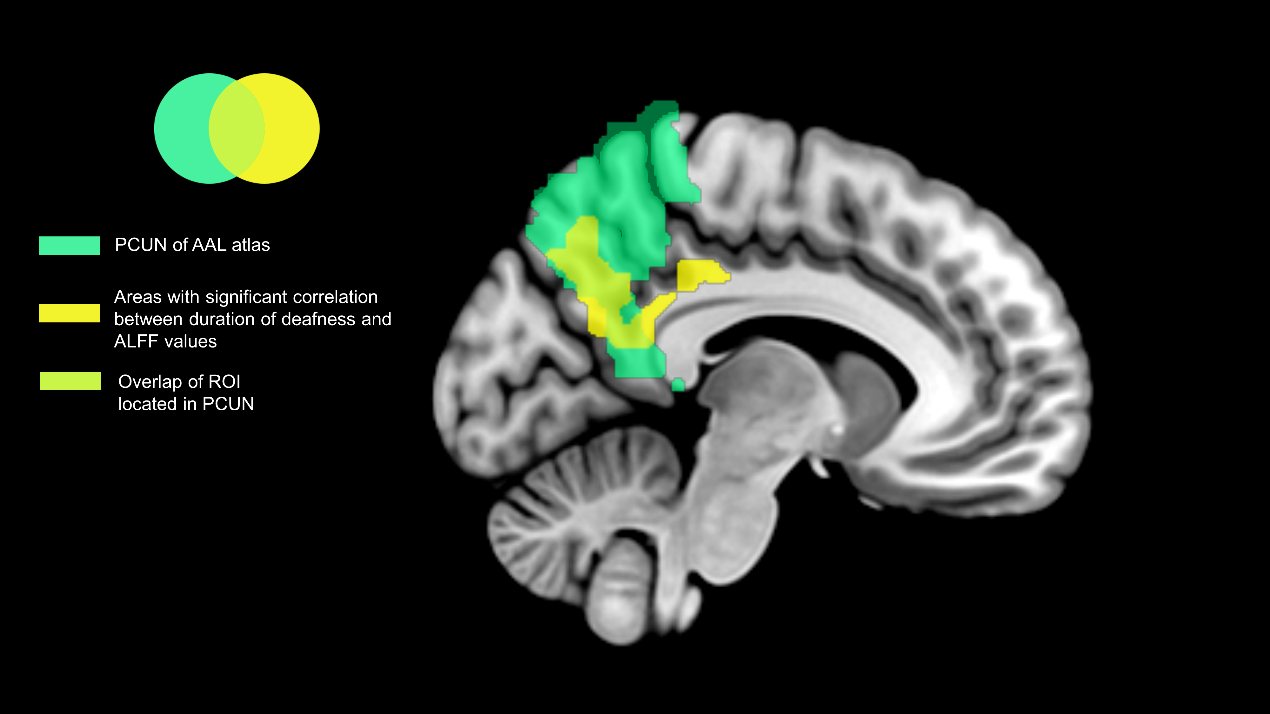
 **Supplementary Figure 1.** ROIs located in PCUN

1. Hypotheses for mediation analyses:

Does ALFF values of PCUN mediate the relation between duration of deafness and SIN threshold?

Mediation hypotheses: Yes, path a, b and a*b are significant

Direct path hypotheses: No, only direct path c′ is significant


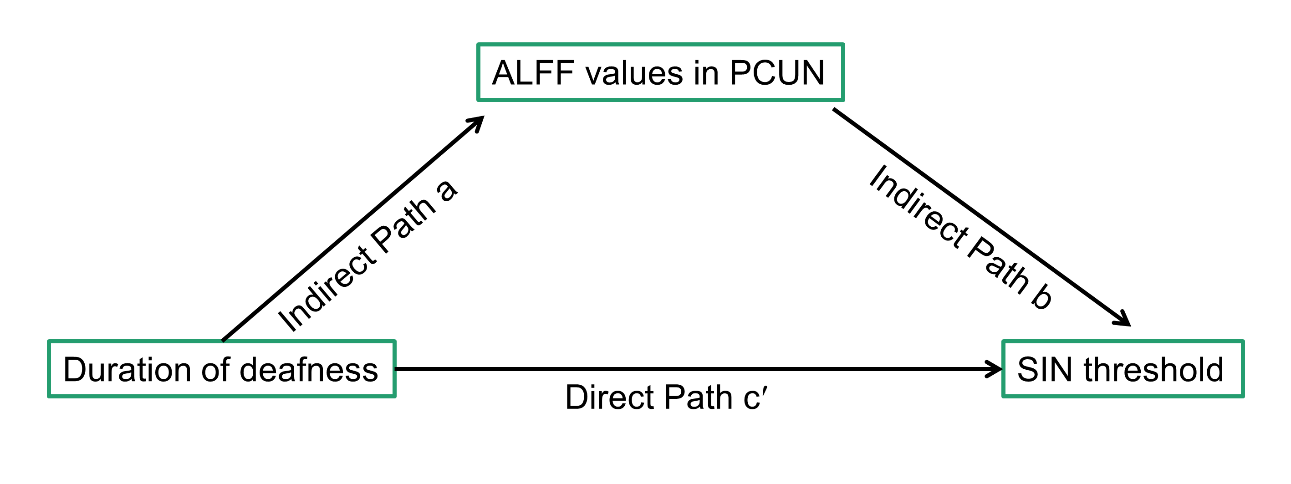
Path diagram with standard notation for path coefficients:

1. Hypotheses for mediation analyses:

Does ALFF values of PCUN mediate the relation between duration of deafness and ASL?

Mediation hypotheses: Yes, path a, b and a*b are significant

Direct path hypotheses: No, only direct path c′ is significant

Path diagram with standard notation for path coefficients:


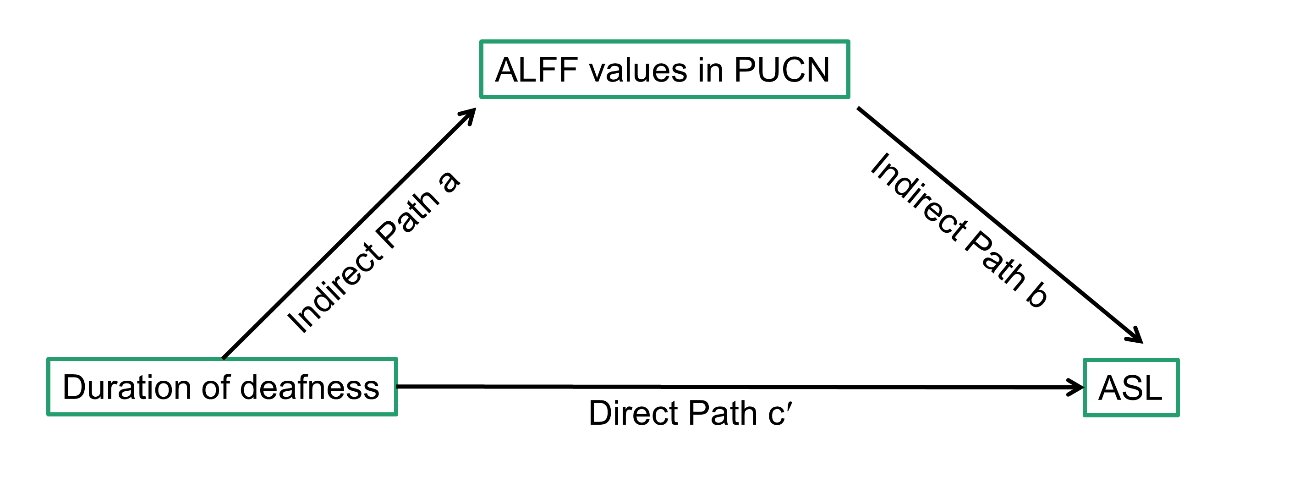


1. Hypotheses for mediation analyses:

Does ALFF values of PCUN mediate the relation between duration of deafness and RMS error?

Mediation hypotheses: Yes, path a, b and a*b are significant

Direct path hypotheses: No, only direct path c′ is significant


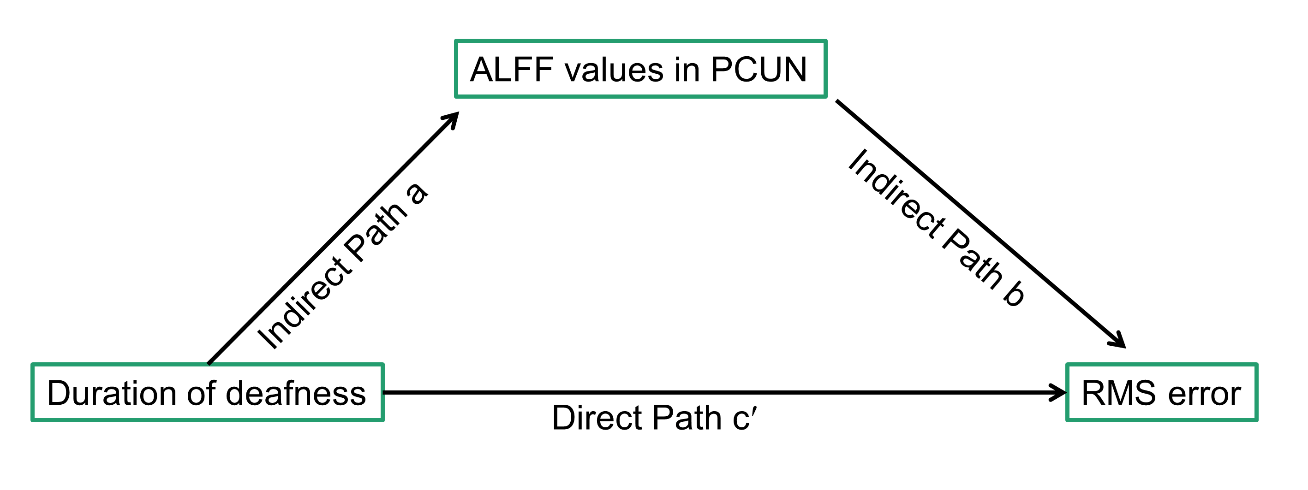
Path diagram with standard notation for path coefficients:

**Results of mediation analysis**

**Supplementary Table 1.** Mediation analysis with the ALFF values of PCUN as the mediator for the relationship between duration of deafness and SIN threshold.

| Predictor Variable |  | Model 1 | | | |  | Model 2 | | | |  | Model 3 | | | |
| --- | --- | --- | --- | --- | --- | --- | --- | --- | --- | --- | --- | --- | --- | --- | --- |
|  |  | β | se | t | p |  | β | se | t | p |  | β | se | t | p |
| Duration of deafness |  | -0.031 | 0.026 | -1.187 | 0.244 |  | 0.020 | 0.004 | 4.805 | <0.001 |  | -0.021 | 0.343 | -0.608 | 0.548 |
| ALFF values in PCUN |  |  |  |  |  |  |  |  |  |  |  | -0.482 | 1.096 | -0.440 | 0.663 |
| R^2^ |  | 0.124 | | | |  | 0.462 | | | |  | 0.129 | | | |
| F |  | 1.503 | | | |  | 9.163 | | | |  | 1.147 | | | |

Model 1, Duration of deafness predicts SIN threshold; Model 2, Duration of deafness predicts ALFF values in PCUN; Model 3, Duration of deafness and ALFF values in PCUN jointly predict SIN threshold.

**Supplementary** **Table 2.** Mediation analysis with the ALFF values of PCUN as the mediator for the relationship between duration of deafness and ASL.

| Predictor Variable |  | Model 1 | | | |  | Model 2 | | | |  | Model 3 | | | |
| --- | --- | --- | --- | --- | --- | --- | --- | --- | --- | --- | --- | --- | --- | --- | --- |
|  |  | β | se | t | p |  | β | se | t | p |  | β | se | t | p |
| Duration of deafness |  | -0.001 | 0.001 | -0.357 | 0.724 |  | 0.020 | 0.004 | 4.805 | <0.001 |  | -0.003 | 0.002 | 1.500 | 0.144 |
| ALFF values in PCUN |  |  |  |  |  |  |  |  |  |  |  | 0.109 | 0.058 | 1.880 | 0.070 |
| R^2^ |  | 0.159 | | | |  | 0.462 | | | |  | 0.245 | | | |
| F |  | 2.019 | | | |  | 9.163 | | | |  | 2.517 | | | |

Model 1, Duration of deafness predicts ASL; Model 2, Duration of deafness predicts ALFF values in PCUN; Model 3, Duration of deafness and ALFF values in PCUN jointly predict ASL.

**Supplementary Table 3.** Mediation analysis with the ALFF values of PCUN as the mediator for the relationship between duration of deafness and RMS error.

| Predictor Variable |  | Model 1 | | | |  | Model 2 | | | |  | Model 3 | | | |
| --- | --- | --- | --- | --- | --- | --- | --- | --- | --- | --- | --- | --- | --- | --- | --- |
|  |  | β | se | t | p |  | β | se | t | p |  | β | se | t | p |
| Duration of deafness |  | -0.053 | 0.278 | -0.190 | 0.850 |  | 0.020 | 0.004 | 4.805 | <0.001 |  | 0.418 | 0.347 | 1.206 | 0.237 |
| ALFF values in PCUN |  |  |  |  |  |  |  |  |  |  |  | -23.211 | 11.064 | -2.098 | 0.044 |
| R^2^ |  | 0.161 | | | |  | 0.462 | | | |  | 0.265 | | | |
| F |  | 2.040 | | | |  | 9.163 | | | |  | 2.793 | | | |

Model 1, Duration of deafness predicts RMS error; Model 2, Duration of deafness predicts ALFF values in PCUN; Model 3, Duration of deafness and ALFF values in PCUN jointly predict RMS error.
